# In-hospital and short-term predictors of mortality in patients with intermediate-high risk pulmonary embolism

**DOI:** 10.34172/jcvtr.2020.51

**Published:** 2020-11-28

**Authors:** Gulay Gök, Mehmet Karadağ, Tufan Çinar, Zekeriya Nurkalem, Dursun Duman

**Affiliations:** ^1^Medipol University Faculty of Medicine, Department of Cardiology, Istanbul, Turkey; ^2^Hatay Mustafa Kemal University Faculty of Medicine, Department of Biostatistics, Hatay, Turkey; ^3^Health Sciences University, Sultan 2. Abdülhamid Han Training and Research Hospital, Department of Cardiology, İstanbul, Turkey

**Keywords:** Acute Pulmonary Embolism, Mortality, Intermediate-High Risk

## Abstract

***Introduction:*** The aim of this study was to evaluate the in-hospital and short-term predictive factors of mortality in intermediate-high risk acute pulmonary embolism (PE) patients with right ventricle (RV)dysfunction and myocardial injury.

***Methods:*** In this retrospective study, the medical records of 187 patients with a diagnosis of intermediate high risk acute PE were evaluated. A contrast-enhanced multi-detector pulmonary angiography was used to confirm diagnosis in all cases. All-cause mortality was determined by obtaining both in hospital and 30 days follow-up data of patients from medical records.

***Results:*** During the in-hospital stay (9.5±4.72 days), 7 patients died, resulting in an acute PE related in-hospital mortality of 3.2%. Admission heart rate (HR), (Odds ratio (OR), 1.028 95% Confidence interval (CI), 0.002-1.121; *P *= 0.048) and blood urea nitrogen (BUN) (OR, 1.028 95% CI, 0.002-1.016; *P *= 0.044) were found to be independent predictors for in-hospital mortality in a multi variate logistic regression analysis. In total, 32 patients (20.9%) died during 30 days follow-up.The presence of congestive heart failure (OR, 0.015, 95%CI, 0.001-0.211; *P *= 0.002) and dementia (OR, 0.029, 95%CI,0.002-0.516; *P *= 0.016) as well as low albumin level (OR, 0.049 95%CI, 0.006-0.383; *P *= 0.049) were associated with 30 days mortality.

***Conclusion:*** HR and BUN were independent predictors of in-hospital mortality and the presence of congestive heart failure, dementia, and low albumin levels were associated with higher 30 days mortality.

## Introduction

Hemodynamically instable patients with acute pulmonary embolism (PE) are classified as high risk group that requires an urgent reperfusion therapy. Albeit, hemodynamically stable patients are classified as intermediate or low risk according to Pulmonary Embolism Severity Index (PESI) score. European Society of Cardiology (ESC) guidelines are further stratifed intermediate risk cases into two groups; as intermediate-high or intermediate-low risk. Intermediate-high risk patients were defined as having both right ventricular (RV) dysfunction and positive cardiac biomarkers in addition to higher PESI score. According to 2019 ESC guidelines, those patients require trombolysis when hemodynamic collaps occurs.^1^ The Pulmonary Embolism Thrombolysis (PEITHO) trial showed that early trombolytic treatment prevented early morality and hemodynamic deterioration in intermediate risk patients, especially those with RV dysfunction and positive for cardiac biomarkers in the expense of an increased risk of bleeding.^2^ Nevertheless, patients with intermediate-high risk acute PE whom may develop hemodynamic collapse or have early death are not evident, and there is a limited data related to outcomes of intermediate-high risk acute PE. In this regard, if we can identify high-risk factors for those patients whom may require intensive monitoring, escalating therapy or early reperfusion, clinical detoriation and early death might be prevented. The aim of this study was to evaluate the in-hospital and short-term predictive factors of mortality in intermediate-high risk acute PE patients who had a RV dysfunction and myocardial injury upon admission

## Materials and Methods

### Study population

In this retrospective study, the medical records of 187 patients who were hospitalized between January 2018 and May 2020 with a diagnosis of acute PE were evaluated. Patients over 18 years old who were hospitalized with the diagnosis of hemodynamically stable acute PE and confirmed by a contrast-enhanced multi-detector pulmonary angiography (CPA) as well as having RV dysfunction, and elevated cardiac biomarkers were included in the study. The exclusion criteria were as the following; I) patients without parameters for the calculation of simple PESI score, II) patients in whom cardiac biomarkers were not measured, III) patients not having RV dysfunction on echocardiography, IV) patients with an simple PESI score of 0, V) patients presented with hemodynamic instability at admission (the presence of cardiac arrest, obstructive shock or persistent hypotension), and VI) patients who did not undergo CPA due to renal failure.

### Data collection

Demographic characteristics, co-morbidities, such as malignancy, hypertension, coronary artery disease, atrial fibrillation, diabetes, congestive heart failure, hyperlipidemia, stroke, dementia, chronic renal insufficiency, hypothyroidism, Parkinson disease (PD) and chronic lung disease as well as physical examination findings, blood gas analysis, laboratory and imaging findings on admission were obtained from medical records. The risk factors for PE, such as smoking, oral contraceptive use, history of immobilization, were also recorded. In addition to clinical, imaging, and laboratory data, the parameters related to aggravating clinical conditions of each patient that were used to calculate simple PESI score were collected.^3^

### In-hospital treatment

In-hospital treatment of each patient was obtained from medical records. All patients received the recommended treatment, as specified in the latest ESC guidelines. Recombinant tissue plasminogen activator (rtPA) at a dose rate of 100 mg IV over 2 hours was used in all patients if hemodynamic detoriation occurs.

Patients with one of the following clinical presentations were considered as having hemodynamic detoriation; I-) development of cardiac arrest, II-) obstructive shock (systolic blood pressure (SBP) <90 mm Hg or vasopressors required to achieve a blood pressure (BP) of >90 mm Hg despite an adequate filling status, in combination with end-organ hypoperfusion), III-) persistent hypotension (SBP < 90 mm Hg or a SBP drop ≥ 40 mm Hg for>15 minutes, not caused by new-onsetarrhythmia, hypovolaemia, or sepsis).^1^

### RV dysfuntion

Patients with at least one of those findings according to echocardiographic examination are considered to having RV dysfunction: I-) RV end-diastolic diameter >30 mm on the parasternal long axis view, II-) the ratio of end-diastolic diameter of RV/the left ventricular end-diastolic diameter >1 on the apical 4-chamber view III-) pulmonary artery pressure greater than 30 mm Hg or with depressed contractility of the RV free wall.^1^

### Myocardial Injury

Myocardial injury was defined as patients having elevated cardiac troponin I (cTnI), which was obtained from venous blood samples at the time of admission. cTnI was measured by using the microparticle enzyme immunoassay (Abbot, USA). cTnI was defined as elevated if greater than 0.05 μg/mL.

### Major and minor bleeding

The major bleeding was defined as; 2 g/dL or more drop in hemoglobin or requiring transfusion of 2 U or more and/or fetal bleeding and/or symptomatic bleeding in a critical organ or area. Otherwise, we defined bleeding as a minor bleeding.^4^

### Study end-points

Mortality was determined by obtaining both in-hospital and 30 days follow-up data of patients from medical records or proxy interviews.

### Statistical analysis

The suitability of the data to normal distribution was tested using the Shaphiro Wilk test. Student t Test was used to compare the parameters with normal distribution, otherwise Mann-Whitney U test was used to compare the parameters without normal distribution. The categorical variables were analyzed by Pearson or Exact Chi-square test. In the study, age, gender, smoking, some clinical characteristics as well as laboratory, treatment and diagnostic results were analyzed firstly with the univariate logistic regression (LR) method, and then the variables found significant were analyzed with the stepwise multivariate LR method (Forward Wald test). By analyzing the significant variables obtained as a result of the last analysis, their performances were examined. Considering the regression co-efficients (β) of the significant variables obtained as a result of the final analysis, their optimal cut-off values were determined by the receiver operation characteristic (ROC) curve analysis. Descriptive statistics are given as mean ± standard deviation for numerical variables and number and % values ​for categorical variables. SPSS Windows version 24.0 package program was used for statistical analysis and *P* < 0.05 was considered statistically significant.

## Results

A total of 187 intermediate-high risk acute PE patients (66.98±14.9 mean age) were included in the study. During in-hospital stay (9.5 ± 4.72 days), 7 patients died, resulting in an APE related in-hospital mortality of 3.2%.In total, 32 patients (20.9%) died during 30 days follow-up. Overall, 108 patients received trombolysis due to hemodynamic impairment. The number of patients recieved trombolysis was higher in the survivor group (62.2% vs. 41%, *P* = 0.017).There were no patients recieving catheter or surgical embolectomy. Of patients treated with thrombolytic, 25.9%(n = 48 patients) had minor bleeding. Major bleeding was not observed. 5.4%(n = 10 patients) of patients need an intravenous inotrop administration and 4.2% (n = 8 patients) required intubation.

Comparison of demographic characteristics, comorbidites, baseline clinical status, length of intensive care unit and in-hospital stay, laboratory and blood gas values on admission between the survivor and non-survivor group were represented in Table 1 and Table 2. Patients in the non-survivor group were older (64.76 ± 15.38 vs. 75.38 ± 9.69, *P*< 0.001) and had more comorbidities (eg.malignancy (23.1% vs. 6.8%, *P* = 0.001), chronic pulmonarydisese (46.2% vs. 18.2%, *P* = 0.001), congestive heart failure (17.9% vs. 4.1%, *P* = 0.002), dementia (7.7% vs. 0.7%,*P* = 0.029), atrial fibrillation (17.9% vs. 3.4%, *P* = 0.001). In terms of laboratory parameters, while blood urea nitrogen (BUN) (43.79 ± 30.5 vs. 30.1 ± 19.2, *P* = 0.002), C-reactive protein (CRP) (117.62 ± 89.1 vs. 59.54 ± 52.8, *P* < 0.001), red cell distribution width (RDW) (16.43 ± 2.66 vs. 15.9 ± 7.08,*P* < 0.001), neutrophile count (10.44 ± 5.12 vs. 8.73 ± 3.84, *P* = 0.020) were significantly higher, albumin (2.87 ± 0.65 vs. 3.44 ± 0.56, *P* < 0.001), sodium (136.64 ± 4.48 vs. 137.64 ± 11.7,*P* = 0.016), folat (5.15 ± 3.21 vs. 9.19 ± 5.07, *P* = 0.015), hemoglobin (11.96 ± 1.97 vs. 13.13 ± 1.96, *P* = 0.001) values were significantly lower.

**Table 1:** Baseline demographics and clinical characteristics of all patients

	**Survivor** **(n=148)**	**Non-survivor** **(n=39)**	***P***
Age, y	64.76±15.38	75.38±9.69	<0.001^a*^
Male, n (%)	59 (39.9)	15 (38.5)	0.873^b^
Diabetes mellitus n, (%)	35 (23.6)	10 (25.6)	0.796^b^
Hypertension, n (%)	87 (58.8)	24 (61.5)	0.755^b^
Atrial fibrillation, n (%)	5 (3.4)	7 (17.9)	0.001^b*^
Smoker, n (%)	62 (41.9)	9 (23.1)	0.041^b*^
Chronic pulmonary disease, n (%)	27 (18.2)	18 (46.2)	0.001^b*^
Coronary artery disease, n (%)	29 (19.6)	12 (30.8)	0.133^b^
Congestive heart failure, n (%)	12 (30.8)	7 (17.9)	0.002^b*^
Malignancy, n (%)	10 (6.8)	9 (23.1)	0.003^b*^
Previous DVT, n (%)	10 (6.8)	1 (2.6)	0.322^b^
Use of OCS, n (%)	3 (2)	0 (0)	0.370^b^
Stroke, n (%)	11 (7.4)	6 (5.4)	0.129^b^
Hypothyroidism, n (%)	14 (9.5)	2 (5.1)	0.530^b^
Chronic renal failure, n (%)	10 (6.85)	5 (12.8)	0.316^b^
Dementia, n (%)	1 (0.7)	3 (7.7)	0.029^b*^
Parkinson disease, n (%)	1 (0.7)	0 (0)	0.493^b^
Hyperlipidemia, n (%)	12 (8.1)	1 (2.6)	0.308^b^
Documented DVT, n (%)	55 (37.2)	17 (43.6)	0.579
HR, beat per minute	102.35±17.69	100.71±21.55	0.384^a^
SBP, mm Hg	127.2±22.13	128.26±21.7	0.761^a^
DBP, mm Hg	75.27±12.83	71.35±14.35	0.202^a^
Temperature, °C	36.64±0.35	36.58±0.3	0.740^a^
Respiratory rate, breaths per minute	19.63±2.84	20±3.6	0.487^a^
O_2_ saturation, (%)	88.69±9.5	88.61±6.11	0.437^a^
Intubation, n (%)	7 (4.8)	1 (2.6)	0.548^b^
Length of hospital stay, days	9.43±4.68	9.74±4.91	0.890^a^
Length of stay in ICC, days	2.44±2.72	3.21±5	0.761^a^

Abbreviations: DVT, deep vein thrombosis; OCS, oral contraceptive use, ICC, intensive care unit; HR, heart rate; SBP, systolic blood pressure; DBP, diastolic blood pressure ^a^ *P* value was obtained from Mann-Whitney *U* test ^b^ *P*value was obtained from Exact and Pearson Chi-square tests *Statistically significant

**Table 2:** Laboratory and echocardiographic findings of all patients

	**Survivor** **(n=148)**	**Non-survivor** **(n=39)**	***P***
Admission blood sugar, mg/dL	164.57±87.27	159.1±71.43	0.848^a^
Uric acid, mg/dL	6.84±2.16	6.62±3.39	0.750^a^
Creatinine, mg/dL	1.06±0.38	1.11±0.39	0.565^a^
GFR, mL/min,	60.81±21.44	56.07±20.13	0.297^a^
BUN, g/dL	30.1±19.24	43.79±30.57	0.002^a*^
AST, U/L	54.17±108.45	51.69±55.7	0.979^a^
ALT, U/L	57.94±158.2	54.59±63.45	0.644^a^
Total bilirubin, mg/dL	0.82±0.55	0.88±0.47	0.256^a^
Direct bilirubin, mg/dL	0.24±0.26	0.31±0.25	0.122^a^
Potassium, mmol/L	5.37±10.92	4.37±0.7	0.439
Sodium, mmol/L	137.64±11.7	136.64±4.48	0.016^a*^
Calcium, mg/dL	8.92±0.79	8.8±0.72	0.099^a^
CRP, mg/dL	59.54±52.85	117.62±89.1	<0.001^*^
Albumin, g/dL	3.44±0.56	2.87±0.65	<0.001^a*^
BNP, pg/mL	4205.79±7716.16	7745.75±12710.08	0.971^a^
T3, ng/dL	2.65±0.52	2.3±0.45	0.006^a*^
T4, ng/dL	1.13±0.26	1.21±0.36	0.459^a^
TSH, μU/mL	1.76±1.79	1.71±1.6	0.784^a^
Vitamin D, pg/mL	18.31±16.29	15.23±11.16	0.559^a^
Vitamin B12, pg/ml	284.45±299.19	353.64±268.12	0.086^a^
Folic acid, μg/dL	9.19±5.07	5.15±3.21	0.015^a*^
Iron, μg/dL	53.4±54.19	30±23.48	0.030^a*^
Iron binding capacity, μg/dL	264.84±101.24	244.42±77.89	0.776^a^
Ferritine, ng/mL	147.22±189.85	132.4±177.21	0.951^a^
WBC, cells/μL	11.56±4.13	12.98±5.3	0.074^a^
Neutrophile, cells/μL	8.73±3.84	10.44±5.12	0.029^a*^
Lymphocyte, cells/μL	1.92±0.89	1.58±0.78	0.048^a*^
Hemoglobin, g/dL	13.13±1.96	11.96±1.97	0.001^a*^
Hematocrit, (%)	40.41±12.04	40.57±27.46	0.009^a*^
RDW, (%)	15.9±7.08	16.43±2.66	0.011^a*^
Platelet, /μL	220.96±87.3	243.28±106.79	0.299^a^
MPV, fL	8.3±1.25	8.18±1.11	0.776^a^
PDW, %	17.59±1.79	17.53±0.65	0.464^a^
D-dimer, ng/mL	5680.56±7165.76	4747±5458.88	0.415^a^
Left ventricle EF, (%)	57.96±6.16	54.68±10.4	0.095^a^
PAP, mm Hg	55.55±14.36	53.68±11.98	0.569^a^
Ph	7.42±0.07	7.4±0.07	0.537^a^
P_co2_, mm Hg	34.76±8.36	38.13±12.01	0.359^a^
P_o2_, mm Hg	51.02±24.5	50.92±21.95	0.975^a^
Lactate, mmol/L	2.72±1.47	2.25±1.08	0.154^a^
Hco_3_, mmol/L	23.0 ±3.99	22.94±3.84	0.984^a^

Abbreviations: GFR, glomerular filtration rate; BUN, blood urea nitrogen; AST, aspartate aminotransferase; ALT, alanine aminotransferase; CRP, c-reactive protein; BNP, brain natriuretic peptide; TSH, thyroid stimulating hormone; WBC, white blood cells; RDW, red cell distribution width; MPV, mean platelet volume, PDW, platelet distribution width; EF, ejection fraction; PAP, pulmonary artery pressure) ^a^ *P* value was obtained from Mann-Whitney *U* test ^b^ *P* value was obtained from Exact and Pearson Chi-square tests *Statistically significant

**Figure 1:**
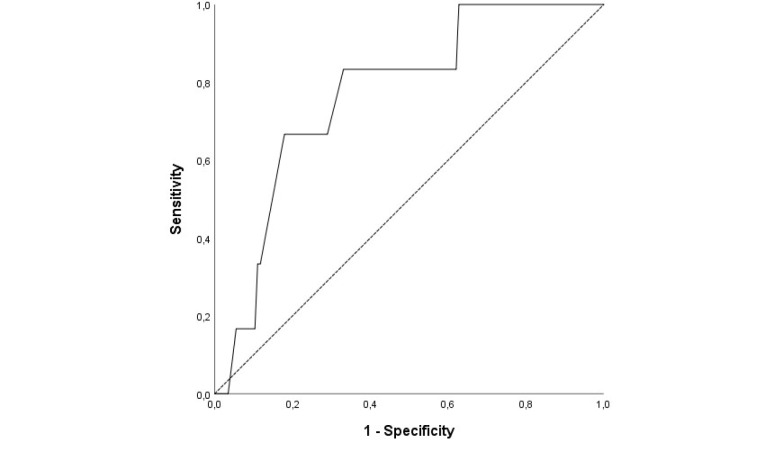
The optimal cut-off point of heart rate for predicting in-hospital mortality

**Figure 2:**
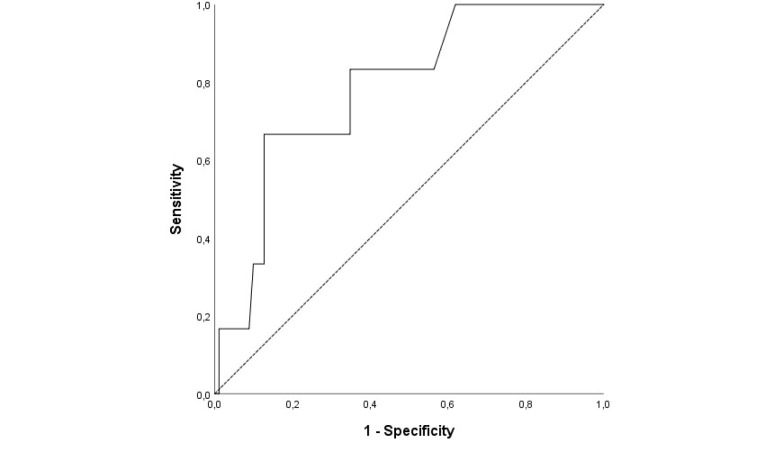
The optimal cut-off point of blood urea nitrogen for predicting in-hospital mortality

HR (OR, 1.028 95% CI, 0.002-1.121; *P* = 0.048) and BUN (OR, 1.028 95% CI, 0.002-1.016;*P* = 0.044) values were found to be independent predictors for in-hospital mortality by multivariate LR analysis (Table 3). As a result of the ROC curve analysis, the optimal cut-off point of HR for in-hospital mortality was > 109.5 (Area under the curve (AUC)= 0.77; 95% CI, 0.61– 0.95; *P* = 0.025) with a sensitivity of 83.3% and a specificity of 67% (Figure 1).The BUN value of 51.5 mg/dL (AUC= 0.78; 95% CI, 0.62– 0.95, *P* = 0.018) was identified as a cut-off point for in-hospital mortality rate, and it had a sensitivity of 66.7%, and a specificity of 87.3% (Figure 2).

**Table 3:** Univariate and multivariate logistic regression analysis for the prediction of in-hospital mortality

	**Univariate LR**		**Multivariate LR**	
	**OR (95%CI )**	***P***	**OR (95%CI)**	***P***
Intubation	295.00 (25.93-3355.73)	<0.001	-	-
CHF	7.727 (1.273-46.915)	0.026	-	-
Heart rate	1.046 (1.001-1.094)	0.047	1.028 (1.001-1.121)	0.048
BUN	1.029 (1.006-1.053)	0.015	1.028 (1.002-1.061)	0.044
MPV	1.642 (1.010-2.669)	0.046	-	-
PAP	1.061 (1.015-1.109)	0.009	-	-
Po_2_	1.039 (1.004-1.076)	0.031	-	-
Lactate	1.488 (0.910-2.433)	0.114	-	-

Abbreviations: CHF, congestive heart failure; BUN, blood urea nitrogen; MPV, mean platelet volume; PAP; pulmonary artery pressure; LR: logistic regression; OR, odds ratio

On univariate analysis; age, the presence of smoking, atrial fibrillation, chronic pulmonary disease, congestive heart failure, malignancy, dementia, BUN, CRP, albumin, T3, neutrophile count, lymphocyte count, hemoglobin, left ventricle ejection fraction, and receiving thrombolysis were found predictors at 30 days mortality (Table 4). Using these predictors, multivariate LR analysis with a forward stepwise selection method was used to predict 30 days mortality. Multivariate LR analysis showed that congestive heart failure (OR, 0.015, 95%CI, 0.001-0.211; *P* = 0.002), dementia (OR, 0.029, 95%CI, 0.002-0.516; *P* = 0.016), and albumin (OR, 0.049, 95%CI, 0.006-0.383; *P* = 0.049 ) were associated with 30 days mortality in intermediate-high risk acute PE patients.

**Table 4:** Univariate and multivariate logistic regression analysis for the prediction of 30 days mortality

	**Univariate LR**		**Multivariate LR**	
	**OR (95%CI )**	***P***	**OR (95%CI)**	***P***
Age	1.067 (1.032-1.104)	<0.001	-	-
Smoking	2.403 (1.065-5.420)	0.035	-	-
Thrombolysis	2.362 (1.150-4.849)	0.019	-	-
Atrial fibrillation	0.160 (0.048-0.536)	0.003	-	-
CPD	0.260 (0.122-0.554)	<0.001	-	-
CHF	0.193 (0.061-0.614)	0.005	0.015 (0.001-0.211)	0.002
Malignancy	0.242 (0.090-0.646)	0.005	-	-
Dementia	0.082 (0.008-0.800)	0.032	0.029 (0.002-0.516)	0.016
BUN	1.023 (1.008-1.038)	0.002	-	-
CRP	1.012 (1.005-1.019)	<0.001	-	-
Albumin	0.155 (0.059-0.403)	<0.001	0.049 (0.006-0.383)	0.049
T3	0.231 (0.079-0.674)	0.007	-	**-**
Neutrophile	1.092 (1.009-1.182)	0.029	-	**-**
Lymphocyte	0.605 (0.382-0.960)	0.033	-	**-**
Hemoglobin	0.752 (0.627-0.901)	0.002	-	**-**
Left ventricle EF	0.950 (0.910-0.992)	0.021	-	**-**

Abbreviations: CPD, chronic pulmonary disease; CHF, congestive heart failure; BUN, blood urea nitrogen; CRP, c-reactive protein; EF, ejection fraction;CI: Confidence interval, LR: logistic regression

**Figure 3:**
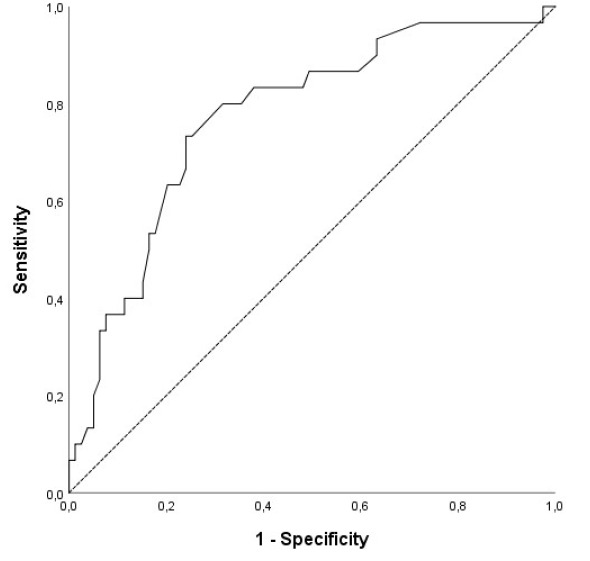
The optimal cut-off point of albumin for predicting 30 days mortality

It was observed that the value of the AUC was statistically significant as a result of the ROC curve analysis on how effective the albumin value in prediction of 30 day mortallity (*P* < 0.001). (The albumin value of <3.17 (AUC = 0.77; 95% CI, 0.67– 0.87*; P* < 0.001) had a sensitivity of 87.7% and a specifity of 59.5%) (Figure 3).

## Discussion

A large number of intermediate-high risk patients with acute PE were recruited in this study. The risk factors found in this study are easy to identify and highly applicable at bedside in clinics, wards, emergency service or intensive care units to predict early death or management strategy of patients whom may require early reperfusion, intensive monitoring or escalating therapy. In our study, comorbidities, such as congestive heart failure and the presence of dementia together were predictors of 30 days mortality but not in-hospital mortality.This finding suggests that patients with intermediate-high risk acute PE and concomitant dementia or congestive heart failure need more attention in the follow-up to prevent early death of those patients.

The independent predictive risk factors of in-hospital mortality were eleveted BUN level and HR.The association of in-hospital mortality with eleveted BUN levels in patients with intermediate to high-risk acute PE may be related to low cardiac output caused by the RV dysfunction. The RV dysfunction decreases the preload or reduces left ventricle filling by shifting to the left of the ventricular septum due to the RV dilatation, which ultimately impairs cardiac output. Low cardiac output compromises renal blood flow and subsequently leads to elevated BUN. Moreover, not only low cardiac output but also venous congestion is related to renal dysfuntion. Venous congestion secondary to RV failure causes central venous pressure to rise. High central venous pressure diminishes renal blood flow, which consequently leads to decreased glomerular filtration rate.^5,6^ The association of mortality with BUN level has been investigated in many studies, including acute decompanseted heart failure, acute coronary syndrome or critically ill patients. In a study,the cut-off BUN level of 34.5 mg/dL was found to be optimal to predict in-hospital mortality for acute PE.^7^ However, in our study, the cut-off value was higher for intermediate-high risk patients with APE. Hospitalized patients with high BUN levels are needed intensive monitoring or escalating therapy to predict early death.

Our results confirmed that combined parameters and clinical findings are suitable for assessment of adverse outcomes, suggesting that HR is helpful to predict in-hospital mortality of those patients.^8,9^ As a result of the ROC curve analysis, the cut-off value of HR > 109.50 was statistically significant (*P* = 0.025) to estimate in-hospital mortality. It was observed that the sensitivity and specificity cut-off values were high enough.

Patients with intermediate-risk acute PE receiving standard anticoagulation therapy have 5.6% incidence of death or hemodynamic detoriation.^2,10^ Early thrombolytic treatment given to those patients prevent early death or hemodynamic decompansation in the expensive of increasing bleeding risk. In our study, the rate of reciving thrombolysis was higher in survivors, which maybe related to that thrombolytic therapy increased survival chance of intermediate-high risk patients with acute PE. Risk stratification for early death in those patients is a crucial step to balance the risk/benefits of early thrombolysis. Accurate identification of patients who are more likely to detoriate is required for advocating escalating therapy (e.g. transfer to the intensive care unit or close monitoring). There are many studies investigating the optimal combined parameters or biomarkers for identifying high risk of patients with acute PE. However, it is still controversial to determine early death or those who are more likely detoriate owing to acute PE.

Albumin has important physiological functions in the endothel stabilization and the inflammatory pathways.^11^ Studies have found that low albumin level disrupts physiological functions of albumin, and it increases susceptibility to tromboembolic events, such as deep venous thrombosis and stroke.^12-14^ Hypoalbuminemi was found to be an independent predictor of mortality in acute PE due to its’ prothrombotic effects, which supports our finding.^11,15,16^ In our study, the value of AUC was statistically significant (*P* < 0.001) for cut-off value of albumin <3.17 to predict 30 day mortality in intermediate-high risk acute PE.

Baseline clinical and laboratory findings of patients with acute PE may not be sufficient to predict mortality.In addition to clinical, imaging, and laboratory parameters, comorbidities, which are related to acute PE severity, are important to assess mortality risk. Various combination of clinical parameters and scores have been built to assesss such mortality. Of those clinical studies, comorbidities, including cancer, chronic lung disease, congestive heart failure, were found to be related to adverse outcomes in patients with acute PE.^17-19^ Patients with congestive heart failure usually have RV dysfunction and this situation worsens with the addition of acute PE. In our study, congestive heart failure was related to high mortality in intermediate-high risk patients with Acute PE-related, which supported this finding.

Acute PE related adverse outcomes were found to be high in normotensive patients who have plasma lactate value of ≥ 2 mmol/L with the combination of RV dysfunction and positive cardac troponins.^20^ However, in our study, plasma lactat levels were not a predictive of mortality for intermediate-high risk acute PE (OR, 0.819 95% CI, 0.385-1.742; *P* = 0.604).

Our study suggests that patients initially defined as intermediate-high risk by either simple PESI score or RV dysfunction or myocardial injury should undergo further risk stratification to identify whom at the highest risk of early death or clinical detoriation and whom may benefit from escalating therapy or early reperfusion. The main advantage of our study was inclusion of a large number of intermediate-high risk patients with APE. The risk factors to predict mortality for these patients are practical, simple, and bed-side use. The applicability of the predictors were easy that can be used in emergency wards or intensive care unit.

The main limitation of our study was that it was a retrospective research. Also, its single center and retrospective design reduced generelazation of the study’s findings. Second, the data dependent on the accuracy of medical records. So, there may be unmeasured or undocumented variables, which might influence on the results. Third, the primary end-point were consisted of either in-hospital death or 30 days mortality. Lastly, patients with hemodynamic collapse were not evaluated.

## Conclusion

In this study, risk stratification of intermediate-high risk acute PE, which was recently defined in the latest ESC guideline, was optimized. Our study suggests that HR and BUN are independent predictors of in-hospital mortality for patients initially defined as intermediate-high risk acute PE. Besides that, the presence of congestive heart failure, dementia and low albumin levels are associated with 30 days mortality in such kind of patients. Besides the simple PESI score, RV dysfunction and positive cardiac biomarkers, we consider that those patients should be evaluated together with their comorbidities and other laboratory parameters to guide for decision-making in acute PE care. However, further studies are required to define which patients might benefit from early thrombolytic treatment witout increasing bleeding risk.

## Competing interests

Not declared.

## Ethical approval

The study was approved by the ethics committee of the Istanbul Medipol University Hospital (10840098-604.01.01-E-14691).

## Funding

None.
